# Maternal urinary triclosan level, gestational diabetes mellitus and birth weight in Chinese women

**DOI:** 10.1016/j.scitotenv.2018.01.102

**Published:** 2018-06-01

**Authors:** Fengxiu Ouyang, Ning Tang, Hui-Juan Zhang, Xia Wang, Shasha Zhao, Weiye Wang, Jun Zhang, Weiwei Cheng

**Affiliations:** a MOE-Shanghai Key Laboratory of Children's Environmental Health, Xinhua Hospital, School of Medicine, Shanghai Jiao Tong University, Shanghai, China; b Departments of Pathology and Bio-Bank, International Peace Maternal and Child Health Hospital, School of Medicine, Shanghai Jiao Tong University, Shanghai, China; c International Peace Maternal and Child Health Hospital, School of Medicine, Shanghai Jiao Tong University, Shanghai, China

**Keywords:** Triclosan (TCS), Prenatal exposure, Gestational diabetes mellitus (GDM), Birthweight, Chinese women

## Abstract

Triclosan (TCS) is an antibacterial chemical widely used in personal-care products and an endocrine disruptor. While TCS exposure is associated with insulin resistance and metabolic disorders in animals, few studies have assessed its effect on the risk of gestational diabetes mellitus (GDM) in humans. This study aimed to explore whether maternal urinary TCS level is associated with the risk of GDM and infant birthweight. We examined 620 pregnant women from Shanghai, China in 2012–2013. Urinary TCS level was measured with high-performance liquid chromatography–tandem mass spectrometry (HPLC–MS/MS), and categorized into high, medium and low in tertiles. GDM was defined based on recommendation of International Association of Diabetes and Pregnancy Study Groups (IADPSG). The GDM rate was 12.7%. TCS was detectable (≥0.1 ng/mL) in 97.9% women (median 2.7 ng/mL). There was a positive, but statistically non-significant association between urinary TCS levels and GDM (adjusted odds ratio 1.17; 95%CI: 0.99, 1.39, with each unit increase of log (TCS) ng/mL) with adjustment for urinary creatinine, maternal age, education, passive smoking, parity and prepregnancy BMI categories. 48.1% of infants were females. Birthweight was 122.8 g higher (95% CI: 13.9, 231.6 g) for female infants of women in high TCS (median 13.3 ng/mL) versus low TCS (median 0.77 ng/mL), with adjustment for urinary creatinine, prepregnancy BMI, GDM and other confounders. No association was found between maternal TCS and birthweight in male infants. These results suggested the potential for TCS to be associated with increased risk of GDM and a gender-specific association with higher birthweight among female infants in a population with widespread but moderate exposure to TCS.

## Highlights

• Triclosan (TCS) was detectable (> 0.1 ng/mL) in 97.9% of Chinese pregnant women.
• Maternal TCS exposure is associated with higher risk of gestational diabetes mellitus, which may partially intermediated via maternal BMI.
• Maternal TCS exposure is associated with higher birthweight among female infants.

## Graphical abstract

**Figure:**
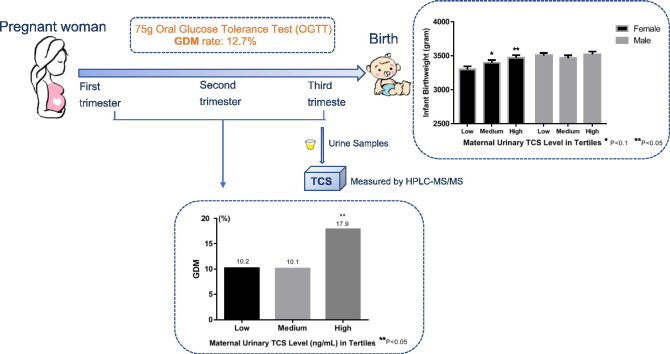

## 1 Introduction

Triclosan (TCS), a broad-spectrum antimicrobial chemical, has been widely used in personal-care products (toothpaste, mouthwash, antibacterial soap, hand sanitizer and cosmetics), clothing and plastics for >40 years (Jones et al., 2000). The common commercial use of TCS has resulted in its ubiquitous presence in the environment, as well as its continuous exposure of various populations (Meeker et al., 2013), including pregnant women (Meeker et al., 2013; Frederiksen et al., 2014; Bertelsen et al., 2014; Casas et al., 2011; Philippat et al., 2012; Wolff et al., 2008; Weiss et al., 2015). In our recent study, 98.2% of urine samples had detectable TCS (≥0.1 ng/mL) (Wang et al., 2017). Absorbed TCS in human body is mainly excreted via urine (Krishnan et al., 2010).

TCS is an endocrine disruptor chemical (EDC) with estrogenic/androgenic and thyroid hormone properties (Stoker et al., 2010; Gee et al., 2008). Animal studies show that TCS exposure induced insulin resistance and metabolic disorder (Regnault et al., 2016). In addition, maternal triclosan exposure was associated with increased plasma glucose, cholesterol, and triglycerides (Rabaglino et al., 2016). There are increasing concerns of EDCs being risk factors for gestational diabetes mellitus (GDM), due to biological plausibility (Ehrlich et al., 2016). The activation of estrogen receptors (ERs) may disrupt energy balance, fat and glucose metabolism (Ropero et al., 2008; Akahori et al., 2008). In addition, TCS was found to be associated with decreased free thyroxine (FT4) levels in women (Geens et al., 2015). The level of FT4 was inversely associated with incidence of GDM (Yang et al., 2016). However, no previous study examined the effect of maternal TCS exposure on GDM (Ehrlich et al., 2016).

In the past two decades, the incidence of GDM has increased dramatically around world (Coustan et al., 2010). The GDM incidence was found to be 18% on average in the Hyperglycemia and Adverse Pregnancy Outcome (HAPO) Study, a large multinational cohort study (Sacks et al., 2012). GDM affects about 18% of pregnancies in China, which has a profound impact on programming offspring metabolic disorders in local population (Yang et al., 2009; Wei et al., 2014). This study aimed to fill in the knowledge gap on whether TCS affects glucose metabolism and occurrence of GDM.

A few studies have examined the association between prenatal TCS and birth weight in industrialized countries where the exposures of TCS were relatively high and the results were inconsistent (Philippat et al., 2012; Wolff et al., 2008). An inverse but non-significant association was found between TCS and birthweight among male, but not female infants in a U.S. study (Wolff et al., 2008). There were a few studies that examined the association between TCS and obesity (Lankester et al., 2013; Li et al., 2015). A recent study reported that urinary TCS level was associated with increased BMI in a U.S. adult population (Lankester et al., 2013).

In this study, we aim to explore whether maternal urinary TCS level is associated with GDM and birth outcomes in male and female infants in China.

## 2 Data and methods

### 2.1 Study population

This birth cohort study was initiated at the International Peace Maternity and Child Hospital (IPMCH), a large tertiary maternity hospital in Shanghai, China, in 2012. Pregnant women were recruited between 2012 and 2013 when they were hospitalized for childbirth. Eligibility criteria included: 1) having routine prenatal care at the study hospitals; 2) singleton pregnancy; 3) planned to reside in Shanghai during the 2-year follow-up period; and 4) willing to participate in this study and sign the consent form. After obtaining a written informed consent, trained nurses conducted a face-to-face maternal interview using a standardized questionnaire and collected spot urine samples. Given that 1/5 to 4/5 of the oral dose of TCS was excreted to urine during the first 4 days after exposure, and the plasma half-life of TCS was 21 h (Sandborgh-Englund et al., 2006), the urinary TCS concentration of the participants should represent their body burdens before hospital admission. The women usually delivered in the next day or two days after our investigation and sample collection. 71.6% of women delivered their infants by cesarean section (CS). After delivery, the study nurses reviewed maternal and infant medical records using a standardized abstraction form to obtain clinical data, including prenatal care, laboratory reports, pregnancy complications, labor and delivery course, and birth outcomes (infant sex, gestational age, birth weight, and birth length). All subjects gave an informed consent.

There were 680 eligible women who were enrolled. For this analysis, we excluded 60 enrolled women because they: did not collect urine sample (n = 38), had a medical complication (syphilitic) (n = 3), conceived by assisted reproductive technologies (ART) (n = 15), or had no urinary creatinine data (n = 1), urinary creatinine concentration < 5 mg/dL (n = 2) (Barbanel et al., 2002) or >300 mg/dL (n = 1) (Barr et al., 2005). This report included 620 women and their infants. 79 women had GDM. Power calculation was based on two-sample (GDM versus non-GDM) *t*-test. If we set significance level (alpha) = 0.05, to detect an effect size 0.35 (i.e. midway between small and medium), the power is 0.83.

This study was approved by the institutional review board of Xinhua Hospital affiliated to Shanghai Jiao Tong University School of Medicine and the International Peace Maternity and Child Hospital. All methods were performed in accordance with the relevant guidelines and regulations.

### 2.2 TCS exposure assessment

The urine samples were stored at −80 °C in polypropylene tubes until they were shipped on dry ice to the Xinhua Hospital, where they were stored at −80 °C before TCS analyses. TCS level was assayed with high-performance liquid chromatography–tandem mass spectrometry (HPLC–MS/MS) analytical method (Agilent 1290–6490, the United State) (Chen et al., 2012). Briefly, 4 mL urine sample was incubated with 2 mL of 1 mol/L ammonium acetate buffer solution (pH = 5.0) for hydrolyzation with 10 μL of β-glucuronidase/sulfatase (20,000 units/mL) at 37 °C overnight. Then the TCS was extracted and preconcentrated with solid phase extraction [500 mg/3 mL, Supelclean ENVI-18, USA]. After drying, the residue was dissolved in methanol. The solution was analyzed by LC-MS/MS. The limit of detection (LOD) was 0.1 ng/mL. The intra- and inter-day CV (coefficient of variation) for TCS were 1.4%–4.6%, and 3.0%–7.4% respectively. The solid phase extraction (SPE) recovery of TCS was 76.9%, and the accuracy (spike) recovery was 88.4%–110%, which indicated that the method is good and reliable. We prepared quality control (QC) samples from spiked pooled urine and analyzed QC samples along with standards, blanks and our urine samples. Creatinine concentrations of urine were measured with an automated chemistry analyzer (7100 Hitachi, Japan).

### 2.3 Outcomes

#### 2.3.1 Main outcomes

##### 2.3.1.1 Definition of GDM

The screening for and diagnosis of GDM followed the recommendation of International Association of Diabetes and Pregnancy Study Groups (IADPSG) (ADA, 2013). Specifically, GDM was defined if a woman had any of the following plasma glucose values: (1) Fasting: ≥5.1 mmol/L; (2) 1 h: ≥10.0 mmol/L; and (3) 2 h: ≥8.5 mmol/L in the 75-g oral glucose tolerance test (OGTT) which was performed at 24–28 weeks of gestation (ADA, 2013). All the diagnoses of GDM were also verified by the obstetricians.

##### 2.3.1.2 Birthweight and Ponderal Index (PI)

Birthweight (g) and birth length (cm) were abstracted from the medical records. PI was calculated as: Birthweight (g) × 100 / Birth length (Frederiksen et al., 2014) (cm^3^).

#### 2.3.2 Secondary outcome

Prepregnancy BMI: Maternal prepregnancy BMI (kg/m (Meeker et al., 2013)) was calculated by prepregnancy weight obtained by self-report during questionnaire interview, and height abstracted from the medical records. We categorized BMI as underweight (<18.5), normal (18.5–22.9), marginal overweight (23–24.9), and overweight (≥25.0) (Ouyang et al., 2009).

### 2.4 Definitions of major covariates

Parity and infant sex were abstracted from the medical records. Maternal socio-demographic factors (including age and education level) and the information of smoking, passive smoking (husband smoking), and drinking during pregnancy were obtained by self-report. The women were asked, “Do you smoke cigarettes during pregnancy?” “If yes, when did you smoke (early-, mid-, or late-pregnancy)?” “How many cigarettes per day did you smoke?” They were also asked if her partner smoked (If yes, “When at home, how many cigarettes per day does he smoke in the room?”).

Gestational age and preterm status: Gestational age was abstracted from the medical records. We determined the gestational age at delivery from maternal last menstrual period (LMP) and early ultrasound (<20 weeks). If the gestational age estimated from the ultrasound differed by >7 days from what was predicted by LMP, then we used the ultrasound assessment. For majority (97.4%) of the women, the gestational age was estimated based on LMP. Preterm was defined as gestational age < 37 weeks, and term as ≥37 weeks.

## 3 Statistical analysis

The TCS levels were categorized into low, medium and high in tertiles. For TCS values below LOD of the assay, a value equal to the LOD divided by the square root of 2 was used for analysis. First, we compared maternal and infant characteristics by tertile of TCS, using ANOVA F-tests for continuous variables and *χ*^2^ tests for categorical data (Table 1). Then, we used multinomial logistic regressions to evaluate the odds of prepregnancy BMI < 18.5, 23–24.9 and ≥25 versus 18.5–22.9 according to prenatal TCS tertiles (eAppendix Tables A). We performed logistic regression to evaluate the association between prenatal TCS tertiles and GDM (Table 2), and used linear regression models to evaluate the associations of TCS with maternal plasma glucose levels during 75 g–OGTT (fasting, at 1 h, at 2 h, respectively), and infant birthweight. The directed acyclic graph is shown at eAppendix Fig. A.

**Table 1:** Characteristics of study participants by maternal prenatal urinary triclosan (TCS) levels.

	TCS (ng/mL)			p value
	Low	Medium	High	
n	206	207	207	
Urinary TCS (ng/mL)				
Median (range)	0.77 (<LOD, 1.44)	2.65 (1.47,4.99)	13.34 (4.99, 95.20)	
log (TCS) (ng/mL)	−0.47 ± 0.75	0.98 ± 0.35	2.77 ± 0.81	<0.001
log (TCS) (μg/g creatinine)	0.48 ± 1.00	1.54 ± 0.78	3.41 ± 1.06	<0.001
Maternal factors				
Age at childbirth (years)	30.2 ± 3.2	30.4 ± 3.4	30.3 ± 3.5	0.77
Height (cm)	162.5 ± 5.0	162.1 ± 4.7	162.1 ± 4.9	0.66
Prepregnancy weight (kg)	54.6 ± 7.2	57.1 ± 9.0	56.4 ± 8.8	0.01
Prepregnancy BMI (kg/m^2^)	20.68 ± 2.48	21.72 ± 3.22	21.45 ± 3.17	0.001
Plasma glucose (mmol/L) during OGTT				
Fasting	4.1 ± 0.5	4.2 ± 0.4	4.3 ± 0.4	0.02
1 h	7.7 ± 1.6	7.8 ± 1.5	8.1 ± 1.9	0.21
2 h	6.4 ± 1.3	6.5 ± 1.2	6.7 ± 1.6	0.21
Length of gestation at time of OGTT (weeks)	26.5 ± 2.5	27.0 ± 2.9	26.3 ± 2.4	0.08
Education				
≤High school	14 (6.9)	10 (4.8)	11 (5.3)	0.32
College	163 (79.9)	181 (87.4)	170 (82.5)	
≥Master degree	27 (13.2)	16 (7.7)	25 (12.1)	
Household income (Chinese Yuan/year)				0.22
<50,000	7(3.4)	9(4.3)	8(3.9)	
50,000–100,000	21(10.2)	25(12.1)	25(12.1)	
100,000–200,000	62(30.1)	49(23.7)	64(30.9)	
>200,000	56(27.2)	66(31.9)	40(19.3)	
Unknown	60(29.1)	58(28.0)	70(33.8)	
Husband smoking during pregnancy	51 (24.9)	54 (26.6)	46 (22.4)	0.62
Parity, nulliparous	182 (88.3)	177 (85.5)	187 (90.3)	0.31
Prepregnancy BMI (kg/m^2^) categories				
<18.5	29 (14.1)	30 (14.5)	34 (16.5)	0.003
18.5–22.9	144 (70.2)	110 (53.1)	116 (56.3)	
23–24.9	22 (10.7)	38 (18.4)	30 (14.6)	
≥25	10 (4.9)	29 (14.0)	26 (12.6)	
GDM	21 (10.2)	21 (10.1)	37 (17.9)	0.03
Hypertensive disorders of pregnancy				
None	183(88.8)	184(88.9)	186(89.9)	0.93
Chronic hypertension or gestational hypertension	15(7.3)	16(7.7)	12(5.8)	
Preeclampsia	8(3.9)	7(3.4)	9(4.3)	
Infant factors				
Gestational age (weeks)	38.7 ± 1.0	38.9 ± 1.2	38.8 ± 1.0	0.19
Birthweight (g)	3414.8 ± 407.3	3429.3 ± 399.4	3497.1 ± 412.0	0.09
Crown-heel length (cm)	50.0 ± 0.8	50.1 ± 0.9	50.2 ± 1.2	0.11
Ponderal Index (100 g/cm^3^)	2.72 ± 0.24	2.72 ± 0.24	2.76 ± 0.24	0.29
Sex, male	114 (55.3)	98 (47.3)	110 (53.1)	0.24
Preterm birth	3 (1.5)	5 (2.4)	0 (0.0)	0.09

Data are presented as Mean ± SD and n (%). OGTT: oral glucose tolerance test; GDM: gestational diabetes mellitus.

ANOVA F-test for continuous variables, chi-square test for categorical values.

**Table 2:** The association between maternal prenatal urinary TCS level (ng/mL) and GDM in 620 women, Shanghai, China.

	Case n (%)	GDM	
		Unadjusted OR (95% CI)	Adjusted^c^ OR (95% CI)
Urinary TCS			
Low	21 (10.2%)	1.00	1.00
Medium	21 (10.1%)	0.99 (0.53,1.88)	0.78 (0.40,1.55)
High	37 (17.9%)	1.92 (1.08,3.41)^b^	1.70 (0.92,3.13)^a^


Trend test	p_trend_ = .02	p_trend_ = .05
Linear log (TCS)	1.20 (1.03,1.41)^b^	1.17 (0.99, 1.39)^a^

a p < .1.

b p < .05.

c Covariates included: log (creatinine), maternal age, education, passive smoking, parity and prepregnancy BMI categories (<18.5, 18.5–22.9, 23–24.9, ≥25 kg/m^2^).

For all regression models, we included natural logarithm of urinary creatinine and those known risk or protective factors of GDM or prepregnancy obesity (maternal age (Montan, 2007), education (Wang et al., 2012), passive smoking (Wang et al., 2012) and parity (Chung et al., 2016)) and prepregnancy BMI categories. For models of birthweight measures as dependent variables (Tables 4), we performed the models in male and female infants separately, and additionally included maternal height, prepregnancy BMI categories, GDM, and gestational age in Model to explore if the associations between TCS and birthweight outcomes go beyond the pathway through maternal obesity and GDM. The approach used for selection of covariates in the final models was to control the potential confounders (eAppendix Fig. A) and gain precision (e.g., the adjustment for maternal height and gestational age for birthweight as dependent variables) (Kleinbaum et al., 1998). All analyses were performed with SAS 9.1 software (SAS Institute, Cary, North Carolina). All reported p values were two-sided, and the level of statistical significance used was p < .05.

## 4 Results

### 4.1 Study population

This study included 620 mother-infant pairs. 48.1% of infants were females. Mean age of the mothers at delivery was 30 (SD 3) years. 94.3% had a college education or above. 24.6% of the women reported exposure to passive smoking (husband smoking). Few women (n = 2) reported smoking, and 2.3% of women reported drinking during pregnancy. We detected TCS in 97.9% of women. 14.6% of women had a BMI of 23–24.9 kg/m^2^, and 10.4% was overweight with BMI ≥ 25 kg/m^2^. 12.7% had diagnosed GDM. The proportion of GDM was similar among women who had CS versus vaginal delivery (13.8% versus 10.2%, *χ*^2^ test, p = .29). No women had pre-pregnancy history of diabetes. Among the three TCS tertile groups, there were differences in maternal prepregnancy weight, prepregnancy BMI (both as continuous and categories), fasting glucose during OGTT, and rate of GDM occurrence (Table 1). For example, the proportion of GDM was about 10% in both the lowest and the medium tertile of urinary TCS, but 17.9% in the highest tertile (*χ*^2^ test, p = .03). There were no associations between maternal urinary TCS and hypertensive disorders (Table 1).

Few (1.3%) infants were born preterm. Mean gestational age was 38.8 (SD 1.1) weeks. 6 infants were born with birthweight <2500 g, and 56 (9%) of infants were born with birthweight ≥4000 g.

### 4.2 TCS, prepregnancy BMI and GDM

With adjustment for urinary creatinine, maternal age, education, passive smoking and parity, the odds of prepregnancy overweight (BMI ≥ 25 kg/m^2^) was 2.69-fold (95% CI: 1.23, 5.91) higher for women with high TCS (median 13.3 ng/mL) versus low TCS (median 0.77 ng/mL) (eAppendix Tables A, Model 3). Every unit increase of natural logarithm value of TCS (ng/mL) was associated with 1.19-fold (95% CI: 0.99, 1.43) higher odds of maternal prepregnancy overweight with adjustments for urinary creatinine and other covariates (eAppendix Tables A, Model 3).

**Fig. 1:**
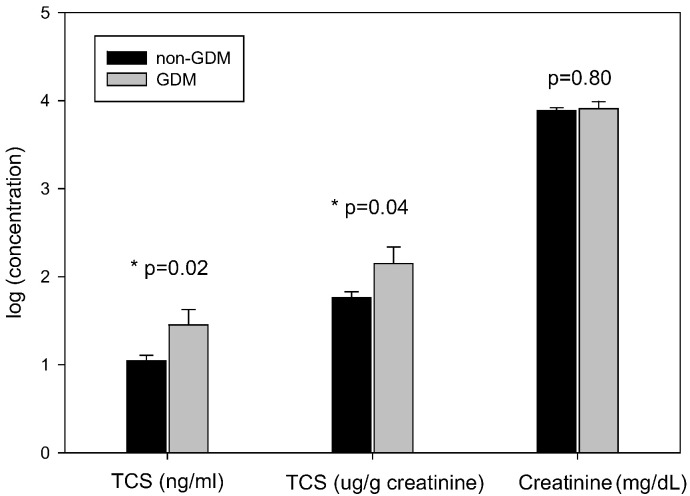
Mean (SE) urinary TCS (ng/mL), TCS (μg/g creatinine), and creatinine (mg/dL) in 79 women with GDM and 541 without GDM, Shanghai, China, 2012 to 2013.

For women with vs. without GDM, as shown in Fig. 1, the natural logarithm of TCS (ng/mL) was 0.41 (95% CI: 0.06, 0.76) higher (Mean ± SD: 1.45 ± 1.55 vs. 1.04 ± 1.47), and the natural logarithm of creatinine-adjusted TCS (μg/g creatinine) was 0.39 (95% CI: 0.02, 0.75) higher in women with GDM (Mean ± SD: 2.15 ± 1.67 vs. 1.76 ± 1.52); but there was no difference in natural logarithm of urinary creatinine concentration (mg/dL) (mean ± SD: 3.91 ± 0.71 vs. 3.89 ± 0.74).

As compared to those with low TCS, women with high TCS levels had higher risk of GDM (OR = 1.70; 95% CI: 0.92, 3.13, p_trend_ = .05) and a higher level of fasting plasma glucose (β = 0.10; 95% CI: 0.01, 0.20 mmol/L, p_trend_ = .04) with adjustment for urinary creatinine, maternal age, education, passive smoking, parity and prepregnancy BMI categories (Table 2, Table 3). Every unit increase of natural logarithm value of TCS (ng/mL) was associated with 1.17-fold (95% CI: 0.99, 1.39) higher odds of GDM, and 0.09 mmol/L higher plasma glucose (95% CI: −0.01, 0.20) at 1 h, 0.07 mmol/L higher (95% CI: −0.01, 0.16) at 2 h in 75 g-OGTT, with adjustments for urinary creatinine, prepregnancy BMI categories and other covariates (Table 2, Table 3), but these associations were not statistically significant.

**Table 3:** The association between maternal prenatal urinary TCS level (ng/mL) and maternal plasma glucose at fasting, 1 h, and 2 h during 75 g-OGTT in pregnant women, Shanghai, China.

	Plasma glucose level during 75 g-OGTT (mmol/L)		
	Fasting	At 1 h	At 2 h
Unadjusted β (95% CI)			
Urinary TCS level			
Low (n = 150)	Ref.	Ref.	Ref.
Medium (n = 127)	0.10 (−0.01,0.20)^a^	0.09 (−0.30,0.49)	0.13 (−0.19,0.45)
High (n = 138)	0.14 (0.04,0.24)^c^	0.34 (−0.05,0.73)^a^	0.29 (−0.03,0.60)^a^
*Trend test*	p_trend_ = 0.006	p_trend_ = 0.09	p_trend_ = 0.08
Linear log (TCS)	0.03 (0.003, 0.06)^b^	0.12 (0.02, 0.23)^b^	0.09 (0.005, 0.18)^b^

Adjusted β (95% CI)^d^			
Urinary TCS level			
Low (n = 150)	Ref.	Ref.	Ref.
Medium (n = 127)	0.04 (−0.06,0.15)	−0.05 (−0.45,0.34)	0.06 (−0.27,0.39)
High (n = 138)	0.10 (0.01,0.20)^b^	0.25 (−0.12,0.63)	0.22 (−0.10,0.54)
*Trend test*	p_trend_ = 0.04	p_trend_ = 0.18	p_trend_ = 0.17
Linear log (TCS)	0.02 (−0.008, 0.05)	0.09 (−0.01, 0.20)^a^	0.07 (−0.01, 0.16)^a^

a p < .1.

b p < .05.

c p < .01.

d Covariates included: log (creatinine), maternal age, education, passive smoking, parity and prepregnancy BMI categories: <18.5, 18.5–22.9, 23–24.9, ≥25 kg/m^2^.

### 4.3 TCS and birth outcomes

For female infants, those born to mothers with high TCS had 122.8 (95% CI: 13.9, 231.6) grams higher birthweight and 0.07 (95% CI: 0.003, 0.13) higher Ponderal Index (100 g/cm^3^) than infants born to mothers with low TCS, with adjustment for urinary creatinine, prepregnancy BMI categories, the presence of GDM and other potential confounders (Table 4, eAppendix Table B). For male infants, maternal TCS was not associated with infant birthweight or Ponderal Index. Sex and TCS effect modification test showed p ≤ .05 for both birthweight and Ponderal Index (Table 4, eAppendix Tables B).

**Table 4:** The association between maternal urinary triclosan levels and birthweight in female and male infants.

	Mean ± SD	Infant birthweight (g)	
		Unadjusted β (95% CI)	Adjusted β (95% CI)^d^
Female			
Urinary TCS (ng/mL)			
Low (n = 92)	3300 ± 422	Ref.	Ref.
Medium (n = 109)	3398 ± 390	97.9 (−12.9, 208.7)^a^	41.9 (−64.8148.5)
High (n = 97)	3471 ± 393	170.2 (56.3, 284.1)^c^	122.8 (13.9231.6)^b^
Trend test		p_trend_ = .003	p_trend_ = .03
Linear log (TCS)		50.9 (18.8, 83.0)^c^	37.2 (6.9, 67.5)^b^

Male			
Urinary TCS (ng/mL)			
Low (n = 114)	3507 ± 372	Ref.	Ref.
Medium (n = 98)	3464 ± 409	−43.2 (−151.5, 65.1)	−88.7 (−190.6, 13.3)^a^
High (n = 110)	3521 ± 428	13.4 (−91.7, 118.4)	−2.0 (−98.5, 94.6)
Trend test		p_trend_ = .81	p_trend_ = .99
Linear log (TCS)		6.1 (−22.2, 34.3)	0.03 (−26.0, 26.1)
p for effect modification^e^		.04	.05

a p < .1.

b p < .05.

c p < .01.

d Covariates included: log (creatinine), maternal age, height, prepregnancy BMI categories, GDM, education, passive smoking, parity and gestational age.

e p value for effect modification estimated in the models with sex (male, female), log (TCS) (linear term) and interaction of the two.

## 5 Discussion

To our knowledge, this is the first study to examine the association between maternal TCS and GDM. We found that maternal urinary TCS was associated with higher risk of GDM in this Chinese urban population in a univariate analysis, but the association was not statistically significant when pre-pregnancy BMI was included in the models. This suggested that triclosan-GDM association may partially intermediated via BMI. In addition, prenatal TCS level was associated with higher birthweight and Ponderal Index in female, but not male infants, after adjusting for potential confounders.

In this study, the urinary TCS concentration of pregnant women was lower than their counterparts in most developed countries. The median TCS was 2.7 ng/mL in this study, lower than what was reported in pregnant women in U.S. (11–24.7 ng/mL) (Wolff et al., 2008; Mortensen et al., 2014), Puerto Rico (26.2 ng/mL) (Meeker et al., 2013), Canada (around 21.6 ng/mL) (Weiss et al., 2015), Spain (6.1 ng/mL) (Casas et al., 2011) and in France (24.1–30 ng/mL) (Philippat et al., 2012), but comparable to or higher than those in Norway (<2.3 ng/mL) (Bertelsen et al., 2014) and Denmark (1.21 ng/mL) (Frederiksen et al., 2014).

The prevalence of GDM among our study participants is similar to the average level in China (Yang et al., 2009). A recent study reported that the prevalence of GDM was 18.9% in Chinese women (Wei et al., 2014). Similarly, the prevalence of GDM was high (12.7%) in this study, and we used criteria of IADPSG for GDM diagnosis (ADA, 2013). In another large-scale study of Chinese pregnant women population, the prevalence of GDM was 4.3% by applying American Diabetes Association (ADA) criteria for GDM diagnosis (Yang et al., 2009). If we used ADA criteria, the prevalence of GDM would be 2.4%. In addition, the prepregnancy BMI of women in this study (mean ± SD: 21.3 ± 3.0 kg/m^2^) is comparable to what was reported in Chinese non-pregnant women of similar age in Shanghai (BMI = 21.1, 95% CI 20.5–21.7 kg/m^2^) (Jia et al., 2002) and in our previous study (BMI: 21.8 ± 2.6 kg/m^2^) (Ouyang et al., 2009).

In this study, high tertile of TCS level was associated with higher risk of GDM and increased levels of plasma glucose at fasting during 75 g-OGTT. We also found that these associations attenuated with additional adjustment for prepregnancy BMI categories. We speculate that the association between TCS and GDM might be explained both by the effect of TCS on maternal overweight, and to a lesser extent, by its direct effect on insulin resistance and glucose intolerance.

The positive association between TCS and risk of GDM is biologically plausible. TCS have estrogenic properties, acting as either an agonist or anagonist to estrogen receptors (Stoker et al., 2010; Gee et al., 2008). The activation of estrogen receptors can affect insulin resistance and glucose metabolism (Ropero et al., 2008; Akahori et al., 2008). ERα stimulates insulin synthesis, and ERβ participates in the regulation of insulin release (Barros & Gustafsson, 2011). Insulin is the only hormone that able to decrease blood glucose (Barros & Gustafsson, 2011). Secondly, the structure of TCS is similar to thyroxine (T4), and the negative association between TCS exposure level and free/or total T4 has been reported in both animal and human studies (Dann & Hontela, 2011; Axelstad et al., 2013). Given that lower FT4 level was associated with higher risk of GDM (Yang et al., 2016), it is evident that higher TCS should be associated with increased risk of GDM. Thirdly, triclosan has recently been found to alter the expression of multiple microRNAs (Huang et al., 2014). The alteration of microRNA expression in placental trophoblast cells may predict the development of GDM (Ehrlich et al., 2016). Also, TCS can be absorbed through ingestion (Sandborgh-Englund et al., 2006), and via skin (Moss et al., 2000). We can't exclude the possibility that the broad-spectrum antibacterial properties of TCS has potential to impact gut microbiota (Pasch et al., 2009). Numerous studies have indicated that gut microbial composition and richness is related to human metabolism (Tehrani et al., 2012).

Few studies have examined the association between TCS exposure and BMI, and the results were inconsistent (Lankester et al., 2013; Li et al., 2015). A recent study of U.S. population aged 20–85 years found that urinary TCS was non-linearly associated with higher BMI, where TCS exposure was relatively high (ranged from <2.3 to 3620 ng/mL) (Lankester et al., 2013). In this pregnant women population of relatively low TCS exposure (range: <0.1 to 95.2 ng/mL), our study suggested that maternal TCS was associated with higher risk of maternal prepregnancy overweight and GDM. Still, future prospective studies are needed.

As for TCS and birthweight, there were a few studies of this topic, and our finding was gender-specific. We found a positive association between maternal TCS and birthweight in female infants and an inverse but non-significant association in male infants. This association cannot be entirely explained by the mediation effect of GDM. Due to lacking of related studies, the pathways that do not involve GDM are yet to be identified. We speculate that the androgenic or estrogenic activity of TCS (Gee et al., 2008) may contribute to the sex modification in the association between TCS and birthweight in this study. In addition, non-association in male infants could be explained by the endocrine disrupting effects of TCS on the placenta (James et al., 2010; Wang et al., 2015). TCS is a potential inhibitor of estrogen sulfonation (James et al., 2010). Thus, high TCS exposure may decrease placental supply of estrogen to fetus, in turn, impair fetal growth (James et al., 2010; Wang et al., 2015). Also, in consistence with the previous studies, TCS was not associated with gestational age in our study (Wolff et al., 2008).

In this study, the rate of CS is high according to its national prevalence (Lumbiganon et al., 2010). This might be due to higher CS rate in tertiary hospitals (Lumbiganon et al., 2010; He et al., 2016). On the other hand, the social and cultural factors (for example, preference for a scheduled day) rather than medical reasons also lead to a higher CS rate in China (He et al., 2016).

The strength of this study is that we were able to collect multiple pre- and perinatal factors, as well as a wide range of anthropometric measures and lab test results, and reach accurate diagnosis of GDM. In addition, the diagnosis of GDM was verified by the obstetricians, thus it ruled out any potential preexisting diabetes that was not identified prior to this pregnancy.

Our study also had limitations. We had a one time measure of prenatal TCS concentration at late pregnancy. However, there were several studies evaluating intra-person correlation and reliability of TCS concentrations in repeated urine samples during pregnancy (Meeker et al., 2013; Bertelsen et al., 2014; Weiss et al., 2015; Philippat et al., 2013), one of which had similar median TCS concentration to ours with an intraclass correlation coefficient (ICC) of 0.49 (Bertelsen et al., 2014). Another recent study also reported that the accuracy was 86.7% when using a single spot urine sample collected during or post-pregnancy to predict the woman's overall TCS concentration corresponding to low, medium, or high exposure level (Weiss et al., 2015). Thus, a spot urine sample was still reasonably good for epidemiologic study to reflect overall TCS exposure during pregnancy (Meeker et al., 2013; Bertelsen et al., 2014; Weiss et al., 2015; Philippat et al., 2013). In addition, any misclassification of TCS exposure levels, if existed, was most likely to be non-differential and draw the association results toward the null. Second, we used creatinine to adjust for urine dilution, and creatinine output rises as pregnancy progresses. However, since the urine samples were collected at similar gestational age with a mean (SD) of 38.8 (1.1) weeks in this study, the influence of creatinine by gestational age on the results should be limited. The use of specific gravity adjustment can be considered in future studies. Thirdly, urinary TCS concentration is relatively low in this study population. Although eligible women were enrolled independent of their GDM status and birth outcomes, caution is needed in generalizing our findings to other populations. Finally, we did not measure other environmental chemicals that may also affect metabolism.

## 6 Conclusion

We found that maternal TCS at environmental exposure level was associated with elevated risk of GDM, but the association did not reach the level of statistical significance. Our results support further prospective study on health effect of maternal TCS exposure in preconception and early pregnancy on mother and their children.

## Sources of financial support

This study was supported by grants from National Natural Science Foundation of China, NSFC [grant number 81673178]; Ministry of Science and Technology of China The National Basic Science Research Program [grant number 2014CB943300], NSFC [grant number No. 81372954] and Shanghai Municipal Education Commission—Gaofeng Clinical Medicine Grant [grant number 20152518], and was partly funded by the Gates Foundation HBGDki project (No. OPP1153191).

## Appendix A Supplementary data

Fig A; eAppendix Table A ; eAppendix Table B Image 1
