# Supplementary material for: Maternal urinary triclosan level, gestational diabetes mellitus and birth weight in Chinese women
Source: Sci Total Environ. 2018 Jun 1;626:451–7. doi: 10.1016/j.scitotenv.2018.01.102 (PMC5849787; doi:10.1016/j.scitotenv.2018.01.102)
Supplement: Supplementary file 1 — Fig A; eAppendix Table A ; eAppendix Table B [file mmc1.doc]

Online Supplemental Materials

| Fig A. Directed acyclic graph illustrating how triclosan (TCS) may be associated with maternal obesity, gestational diabetes mellitus (GDM) and infant birthweight.  **GDM**  **Maternal Obesity**  **Infant Birthweight**  **Ponderal Index**  **Infant sex, Gestational age, Maternal height**  **Other factors: Maternal age, education, passive smoking, parity et al.**  **TCS**  (Prepregnancy BMI) |
| --- |
| eAppendix Table A. The association between maternal prenatal urinary TCS level (ng/mL) and prepregnancy overweight in 620 women, Shanghai, China.   |  |  | OR (95% CI) |  | | --- | --- | --- | --- | |  | Prepregnancy BMI < 18.5 | 23-24.9 | >25 | | Urinary TCS | **Model 1: Unadjusted** | | | | Low | 1.00 | 1.00 | 1.00 | | Medium | 1.35 (0.77,2.39) | 2.26 (1.27,4.04)^c^ | 3.80 (1.77,8.12) ^c^ | | High | 1.46 (0.84,2.53) | 1.69 (0.93,3.09)^a^ | 3.23 (1.50,6.97) ^c^ | | *Trend test* | P_trend_=0.18 | P_trend_=0.09 | P_trend_=0.004 | | Linear log (TCS) | 1.06 (0.91, 1.23) | 1.18 (1.01,1.38)^b^ | 1.25 (1.05, 1.49)^b^ | |  | **Model 2: Model 1 + log (creatinine)** | | | | Low | 1.00 | 1.00 | 1.00 | | Medium | 1.48 (0.83,2.64) | 1.95 (1.08,3.53)^b^ | 3.15 (1.45,6.84) ^c^ | | High | 1.58 (0.90,2.77) | 1.45 (0.78,2.68) | 2.71 (1.24,5.92) ^c^ | | *Trend test* | P_trend_=0.11 | P_trend_=0.27 | P_trend_=0.02 | | Linear log (TCS) | 1.08 (0.93, 1.26) | 1.13 (0.96,1.33) | 1.19 (0.99, 1.42)^a^ | |  | **Model 3: Model 2+covariates**^d^ | | | | Low | 1.00 | 1.00 | 1.00 | | Medium | 1.44 (0.79,2.60) | 1.86 (1.02,3.40)^a^ | 3.04 (1.39,6.66) ^c^ | | High | 1.61 (0.91,2.86) | 1.45 (0.78,2.68) | 2.69 (1.23,5.91) ^c^ | | *Trend test* | P_trend_=0.10 | P_trend_=0.27 | P_trend_=0.02 | | Linear log (TCS) | 1.09 (0.93, 1.27) | 1.12 (0.95, 1.32) | 1.19 (0.99, 1.43)^a^ | | ^a^p<0.1. ^b^p<0.05. ^c^p<0.01  ^d^Covariates included: maternal age, education, passive smoking and parity. | | | | |

| eAppendix Table B. The association between urinary triclosan levels and infant Ponderal index. | | | | |
| --- | --- | --- | --- | --- |
|  | Ponderal Index (100 gram/cm^3^)  β (95% CI) | | | |
|  | Mean ± SD | Model 1: Unadjusted | Model 2:  Model 1+log (creatinine) | Model 3:  Model 2+ covariates^d^ |
| **Female** |  |  |  |  |
| Urinary TCS (ng/mL) |  |  |  |  |
| Low (n=92) | 2.66±0.24 | Ref. | Ref. | Ref. |
| Medium (n=109) | 2.71±0.23 | 0.05 (-0.01, 0.12)^a^ | 0.04 (-0.02, 0.10) | 0.02 (-0.04, 0.09) |
| High (n=97) | 2.75 ± 0.21 | 0.09 (0.03, 0.16) ^c^ | 0.08 (0.01, 0.14)^b^ | 0.07(0.003, 0.13)^b^ |
| *Trend test* |  | P_trend_=0.005 | P_trend_=0.02 | P_trend_=0.04 |
| Linear log (TCS) |  | 0.03 (0.01, 0.05) ^c^ | 0.02 (0.01, 0.04) ^c^ | 0.02 (0.003, 0.04)^b^ |
| **Male** |  |  |  |  |
| Urinary TCS (ng/mL) |  |  |  |  |
| Low (n=114) | 2.78±0.24 | Ref. | Ref. | Ref. |
| Medium (n=98) | 2.74±0.26 | -0.04 (-0.11, 0.03) | -0.05 (-0.12, 0.02) | -0.06 (-0.13, 0.003)^a^ |
| High (n=110) | 2.76±0.27 | -0.01 (-0.08, 0.05) | -0.02 (-0.09, 0.04) | -0.03 (-0.09, 0.04) |
| *Trend test* |  | P_trend_=0.66 | P_trend_=0.52 | P_trend_=0.45 |
| Linear log (TCS) |  | -0.0001 (-0.02, 0.02) | -0.002 (-0.02, 0.02) | -0.004 (-0.02, 0.01) |
| P for effect modification^e^ |  | 0.03 | 0.04 | 0.04 |
| ^a^p<0.1. ^b^p<0.05. ^c^p<0.01  ^d^Covariates included: maternal age, height, prepregnancy BMI categories, GDM, education, passive smoking, parity and gestational age.  ^e^p value for effect modification estimated in the models with sex (male, female), log (TCS) (linear term) and interaction of the two. | | | | |
